# Transient diabetes insipidus in post CABG patient after vasopressin withdrawal: A report

**DOI:** 10.6026/973206300213564

**Published:** 2025-10-31

**Authors:** Mukesh Kumar Jaswant Singh Pachahara, Rajat Kalra, Ravi Roshan, Hirday Kumar

**Affiliations:** 1 Department of Anesthesia, Narayan Medical College & Hospital, Sasaram, Bihar, India; 2 Department of Cardiothoracic and Vascular Surgery, Narayan Medical College & Hospital, Sasaram, Bihar, India

**Keywords:** Transient diabetes insipidus, post CABG, vasopressin withdrawal

## Abstract

Vasopressin is increasingly being used as inotropic agent due to its catecholamine's sparing property. Vasopressin is instrumental in rapid adjustment of water excretion according to the state of body hydration. It is also used in treatment of DI; Arginine vasopressin disorder, formerly known as diabetes insipidus (DI), is a disease process that results in either decreased release of antidiuretic hormone (Anti Diuretic Hormone (ADH), also known as vasopressin or Arginine Vasopressin (AVP) or reduced response to ADH, causing electrolyte imbalances. however, there are only anecdote reports discussing the precipitation of DI d/t vasopressin withdrawal. Hence, we report a case of diabetes insipidus in post Coronary artery bypass grafting after vasopressin withdrawal.

## Case report:

A 68yr/Male admitted with c/o Chest pain while walking since 11/2 year with pain radiating to left hand & unable to lift left hand with Dyspnea on exertion. Patient was taken for coronary artery bypass grafting on pump, CABG was done on arrested heart with left internal mammary artery grafted to Left anterior descending artery & 2 reverse saphenous graft used to bypass significant stenosis in obtuse marginal-1 & mid right coronary artery. Patient was weaned from Cardio pulmonary bypass pump on Dopamine 5mcg/kg/min, Milrinone 0.25mcg/kg/min, Vasopressin 0.02units/kg/hr , patient was extubated on POD-1 & Inotropes weaning was started, Vasopressin was tapered 1st over a period of 10hrs, However after stopping vasopressin; Urine output increased significantly to 3ml/kg/hr from 1ml/kg/hr. Other causes of polyuria like Hypercalcemia, Hypokalemia, and Hyperglycemia were ruled out, Polyuria resulted in significant decrease in CVP from 12cmofH2O to 4cmofH2O & volume replacement was needed to avoid hypotension and Hyponatremia due to dehydration, Sodium levels was corrected accordingly. MRI head revealed - no cerebral insult, Ultrasonography of KUB was within normal limits, KFT: within normal limits, Urine workup showed decrease in Urine osmolarity & (1.005) specific gravity. Nephrology opinion was taken and opined about Diabetes Insipidus probably due to vasopressin withdrawal. POD4 vasopressin was reintroduced @0.02IU/KG/HR which showed therapeutic response in the urine output to 1.5ml/kg/hr. Tab.Desmopressin was started after failure to wean vasopressin, thereafter over 72hrs Vasopressin was weaned off successfully, patient was discharged on POD-10 on oral T. Desmopressin along with T. Ecosprin, T. Metoprolol & Statins.1 month after Sx, T. Desmopressin was weaned off successfully.

## Salient points/discussion:

Vasopressin is secreted by posterior pituitary gland [1, 2]. Vasopressin the ADH is the main hormone that regulates body fluids Osmolality, it also plays vital role in physiological functions such as vasoconstriction, water retention & corticotropin secretion etc. Vasopressin receptors are also expressed in vascular smooth muscle cell resulting in vasoconstriction & increasing SVR. Vasopressin also increases level of catecholamines in receptors merely resulting in decreased dosage requirement. Destruction of Neurons in or near the supraoptic & paraventricular Nuclei of the Hypothalamus from pituitary surgery, Trauma, cerebral Ischemia or Malignancy may cause central Diabetes Insipidus from lack of vasopressin release. There is also possibility of disturbances in nonosmotic stimulus pathway from left atrium which communicate with the hypothalamus through the vagus parasympathetic stimulus. The manipulation of heart for grafting can cause relative left atrial hypovolemia and suppression of ADH release [3]. Diabetes Insipidus is a disorder of water metabolism associated with Polyuria, Urine hypotonicity & hypernatremia. The quantitative criteria include urine output greater than 200ml/hr or 3ml/kg/hr, urine osmolality less than 200mosm/KG & plasma sodium greater than 145meq/L. It can be Neurogenic or Nephrogenic central DI which is characterized by a lack of ADH. Clinical management of DI consists of replacing body water deficits and stopping losses, which can be done by administering hypotonic fluid and exogenous VP or an analog, such as D-arginine-vasopressin (desmopressin, DDAVP) [4, 5]. Vasopressin is increasingly being used in cardiac Sx due to its catecholamine sparing effect & increasing SVR without increasing Heart rate.Urine output varied significantly over the postoperative period, peaking on the first postoperative day(11,000 ml) before gradually declining on subsequent days (Figure 1 - see PDF).

## Conclusion:

Cardiac Surgeries done on Cardio pulmonary bypass has been shown to cause a six fold increased circulating ADH levels 12 hrs after surgery. In some cases, ADH release may be transiently suppressed due to Cardioplegia or CPB leading to DI. DI should be considered in individuals who have undergone Open heart surgery & develop polyuria; When DI is diagnosed adequate ADH administration may prevent further serious problems like post-operative cerebral Ischemia.
